# Estimating the lifelong health impact and financial burdens of different types of lung cancer

**DOI:** 10.1186/1471-2407-13-579

**Published:** 2013-12-05

**Authors:** Szu-Chun Yang, Wu-Wei Lai, Wu-Chou Su, Shang-Yin Wu, Helen HW Chen, Yi-Lin Wu, Mei-Chuan Hung, Jung-Der Wang

**Affiliations:** 1 Department of Internal Medicine, National Cheng Kung University Medical College and Hospital, 138 Sheng Li Road, Tainan 70403, Taiwan; 2 Department of Public Health, College of Medicine, National Cheng Kung University, No.1 University Road, Tainan 70101, Taiwan; 3 Department of Surgery, National Cheng Kung University Medical College and Hospital, 138 Sheng Li Road, Tainan 70403, Taiwan; 4 Department of Radiation Oncology, National Cheng Kung University Medical College and Hospital, 138 Sheng Li Road, Tainan 70403, Taiwan; 5 Department of Nursing, National Cheng Kung University Medical College and Hospital, 138 Sheng Li Road, Tainan 70403, Taiwan

**Keywords:** Lung cancer prevention, Life expectancy, Quality-adjusted life expectancy, Costs, Out-of-pocket money

## Abstract

**Background:**

Owing to the high mortality and rapidly growing costs related to lung cancer, it is worth examining the health benefits of prevention for major types of lung cancer. This study attempts to quantify the quality-adjusted life expectancy (QALE), loss-of-QALE, and lifetime healthcare expenditures of patients with different pathological types of lung cancer.

**Methods:**

A national cohort consisting of 66,535 patients with pathologically verified lung cancer was followed for 13 years (1998–2010) to obtain the survival function, which was further extrapolated to lifetime. Between 2011 and 2012, EuroQol 5-dimension questionnaires were used to measure the quality of life (QoL) for 1,314 consecutive, cross-sectional samples. After multiplying the lifetime survival function by the utility values of QoL, we estimated the QALE and loss-of-QALE. We also collected the monthly healthcare expenditures, which included National Health Insurance-reimbursed and out-of-pocket direct medical costs, for 2,456 patients from 2005 to 2012. These values were multiplied by the corresponding survival probabilities to calculate lifetime healthcare expenditures after adjustments with medical care inflation rates and annual discount rates.

**Results:**

The QALE for patients with small cell lung cancer, squamous cell carcinoma, and adenocarcinoma were 1.21, 2.37, and 3.03 quality-adjusted life year (QALY), with the corresponding loss-of-QALE of 13.69, 12.22, and 15.03 QALY, respectively. The lifetime healthcare expenditures were US$ 18,455 ± 1,137, 20,599 ± 1,787, and 36,771 ± 1,998, respectively.

**Conclusions:**

The lifelong health impact and financial burdens in Taiwan are heavier for adenocarcinoma than for squamous cell carcinoma. The cost-effectiveness of prevention programs could be directly compared with that of treatment strategies to improve patient value. And the methodology could be applied to other chronic diseases for resources planning of healthcare services.

## Background

Over the past two decades, mortality attributed to lung cancer has increased [1], and it has become the leading cause of cancer deaths [1,2]. Owing to the development of new interventions to diagnose and treat lung cancer, lung cancer care costs are continuously rising [3]. Therefore, healthcare providers are now facing an increased burden of caring for lung cancer patients. It is thus worth examining the health benefits of prevention for major types of lung cancer.

For assessing health benefits, both survival and quality of life (QoL) should be taken into consideration, and quality-adjusted life expectancy (QALE) using quality-adjusted life year (QALY) as the unit may be more suitable than estimating survival alone for the purpose of comparison of various types of healthcare services [4-6]. Previous studies mostly have focused on cross-sectional analyses of the economic burden of lung cancer [7,8]. However, lifetime medical costs should be estimated to evaluate the cost-utility of prevention programs. Although almost all cancer-specific medical costs are reimbursed by the National Health Insurance (NHI) available in Taiwan, there are out-of-pocket medical costs that must be estimated to obtain the lifetime healthcare expenditures attributable to lung cancer. Transportation costs, payments to caregivers, home adaptation due to illness and human capital loss were not taken into consideration in this analysis.

Since treatments and prognoses for different pathological subtypes of lung cancer are different, we hypothesized that they may impact mortality, QoL, and medical costs to different degrees. Based on data from the National Cancer Registry, NHI reimbursements, and National Cheng Kung University Hospital (NCKUH), this study is aimed at quantifying the QALE, loss-of-QALE and lifetime healthcare expenditures that occur in patients with different subtypes of lung cancer that would be regained through successful prevention initiatives.

## Methods

The Institutional Review Board of NCKUH approved this study before its initiation (Approval number: ER-100-079), and every patient interviewed provided written informed consent. This study abstracted data from the National Cancer Registry for survival analysis, combined this with the national life tables to extrapolate the survival function to lifetime, collected QoL and costs data from lung cancer patients in NCKUH, and integrated the survival function with the QoL and costs to estimate the life expectancy, QALE, loss-of-QALE, and lifetime healthcare expenditures per case of lung cancer. QALE was estimated using the following equation [9-11]:

$$\mathrm{QALE}=\int E\left[\mathrm{QoL}\left(t/x\right)\right]S\left(t/x\right)\mathrm{dt}$$

**Figure 1:**
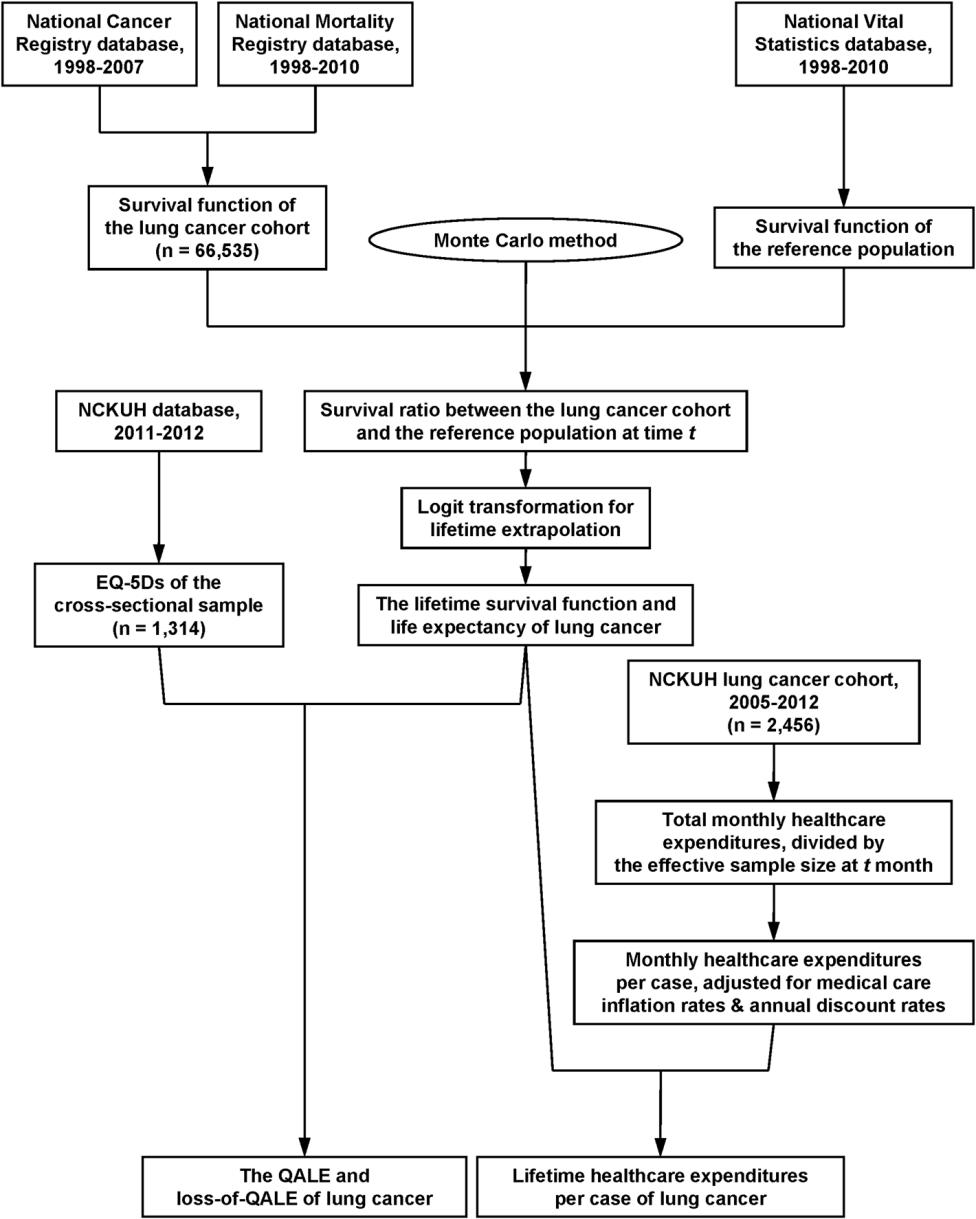
**Flow diagram of the inclusion of subjects and their relevant information for estimation.** EQ-5D: EuroQol 5-dimension questionnaire; NCKUH: National Cheng Kung University Hospital; QALE: quality-adjusted life expectancy.

*E*[*QoL(t/x)*] denotes the expected value of the QoL function for condition *x* at time *t,* and *S*(*t/x*) denotes the survival function for condition *x* at time *t*. The entire design is summarized in Figure 1.

### National Cancer Registry for estimation of the survival function

All patients with lung cancer during the period from 1998 to 2007 were abstracted from the National Cancer Registry database. The diagnosis of lung cancer and its subtypes was based on either histology or cytology codes taken from the International Classification of Diseases [12]. The survival status for each patient was verified by linking the patient’s identification information to the National Mortality Registry database. Each patient underwent follow-up from the day of diagnosis until the end of 2010.

### Extrapolating the survival to lifetime

After obtaining the survival function of the lung cancer cohort, a method proposed by Huang and Wang [13] was used to extrapolate this curve beyond the end of the follow-up period. This approach assumes that lung cancer generates a constant excess hazard (*i.e.*, mortality) after a certain follow-up period, and its calculation is carried out in three steps. First, we took the hazard functions from the life tables of the National Vital Statistics of Taiwan to generate an age- and sex-matched reference population using the Monte Carlo method and then estimated the survival function. Second, we calculated the survival ratio between the lung cancer cohort and the reference population at each time *t* and performed a logit transformation of this. Third, the logit transformation of the ratio was fitted by simple linear regression up to the end of the follow-up period. The estimated regression line, together with the survival function of the reference population beyond the follow-up limit, was used to extrapolate the lifetime survival function of the lung cancer cohort, and thus the life expectancy of the lung cancer cohort (up to 600 months) after diagnosis could be estimated. Expected years of life lost in the lung cancer cohort were defined as the survival difference between the cohort and the reference population. The method described above has been demonstrated to be effective using computer simulations [13], proven mathematically [14], and corroborated by several examples of cancer cohorts [15,16]. The iSQoL statistical package [17] was used to facilitate the computation. To validate the extrapolation method described above, we used the survival data of patients who were diagnosed during the first seven years and then extrapolated them to 13 years. Because these patients were actually followed until the end of 2010, the mean survival duration within the 13-year follow-up period, using the Kaplan-Meier method, was considered as the gold standard. The relative bias was computed to compare the difference in values between the extrapolation and the Kaplan-Meier estimation.

### Measuring the QoL from a cross-sectional sample

From May 2011 to April 2012, all consecutive patients with lung cancer from the outpatient oncology, chest surgery, and chest medicine departments of NCKUH were invited to participate in this study. To enrich our sample, we also screened patients admitted to the wards between November 2011 and January 2012. The inclusion criteria included the realization of a lung cancer diagnosis in the participants, the absence of malignancy at another site, and the subjects’ ability to understand and answer the questionnaire. In some individuals, measurements were performed repeatedly; however, each measurement took place 3 months apart.

The EuroQol 5-dimension questionnaire (EQ-5D) [18,19], which is a preference-based generic instrument, was used to estimate the QoL utility values. The five dimensions assessed by EQ-5D are mobility, self care, usual activities, pain/discomfort, and anxiety/depression, each with three levels of severity. Using the scoring function from the United Kingdom [20], these health state parameters were transformed into a utility value ranging from 0 to 1, in which 0 represents death and 1 indicates perfect health.

The duration-to-date for each measurement was defined as the period between the date the lung cancer diagnosis was made and that of the interview. A kernel-smoothing method (i.e., the moving average of the nearby 10%) was used to estimate the mean QoL function [10,11]. The QoL utility values beyond the follow-up period were assumed to be the same as the average of the last 10% of patients near the end of follow-up.

### Estimating the QALE and loss-of-QALE

The lifetime survival function of the lung cancer cohort was adjusted using the corresponding mean QoL function to obtain a quality-adjusted survival curve, in which the sum of the area under the curve was the QALE of the lung cancer patients [10]. The loss-of-QALE was calculated by subtracting the area under the quality-adjusted survival curve of the lung cancer patients from that of the reference population. Because the EQ-5D scoring function for Taiwanese residents is still under development, we tentatively assumed the QoL score of the reference population to be 1 [10,13]. However, we also tested the assumption in sensitivity analysis assuming the average QoL values of 0.9 and 0.95 for the reference population. We used the iSQoL software [17], which can be downloaded for free, to facilitate the computation.

### Estimating the lifetime healthcare expenditures

The reimbursement database at NCKUH was used to obtain spending details on medical services for lung cancer patients between January 2005 and December 2012. As these data included not only medical costs paid by the NHI but also out-of-pocket money, most direct medical costs attributable to lung cancer could thus be obtained to calculate the total monthly healthcare expenditures. These were then divided by the effective sample sizes, namely, the number of lung cancer patients who survived that month, to obtain the average monthly healthcare expenditures per case. These values were subsequently multiplied by the corresponding monthly survival probabilities and summed up to obtain the lifetime healthcare expenditures per case. All the payments in different calendar years were adjusted based on the related consumer price indices [21] and made equivalent to those in 2012. To discount costs in future years, we also adjusted the healthcare expenditures, using an annual discount rate of 3%. Because patients might incur expenses outside the hospital, we compared the costs we estimated with the costs reimbursed in the national database by using the same group of patients (2005–2007). The proportion of reimbursement at NCKUH was estimated. A total of 1,002 patients ever cared at the NCKUH were linked with the reimbursement database of NHI, of which 11 cases could not be successfully connected. There were 4 cases (2 cases of small cell lung cancer (SCLC) and 2 cases of non-small cell lung cancer (NSCLC)) with the difference on the date of diagnosis larger than 1,000 days and were not included for the above calculation.

## Results

The lung cancer cohort from the National Cancer Registry database used to obtain the survival function consisted of 66,535 patients. A total of 964 lung cancer patients visited NCKUH between 2011 and 2012, and 676 patients met the criteria for inclusion. Individuals who declined to answer the questionnaire were excluded, leaving 635 patients. However, 17 of these had incomplete data, and thus the cross-sectional sample for measuring the QoL consisted of a total of 618 patients, and 1,314 QoL measurements were performed. The average number of measurements per person was 2.1. In addition, the reimbursement database in NCKUH included details of healthcare expenditures for 2,456 lung cancer patients from 2005 to 2012. Table 1 summarizes the descriptive statistics of the national cohort for survival function and the NCKUH cohort for healthcare expenditures. By using the same method, patients with NSCLC in the NCKUH cohort were found to have longer life expectancy and greater NHI-reimbursed lifetime healthcare expenditures than those in the national cohort, which can be partially explained by the advancement of NSCLC treatment in the past five years and different frequency distributions of pathological subtypes in the two groups.

**Table 1:** Comparison between the national cohort and the cohort from National Cheng Kung University Hospital (NCKUH)

	**The national cohort**	**The NCKUH cohort**	***P *** value^*^
Calendar years of collection	1998-2007	2005-2012	
Total number of patients	66,535	2,456	
Age at diagnosis, mean (SD) years	67.22 (12.10)	65.57 (12.57)	
Sex, no. of males (%)	45,098 (67.78)	1,457 (59.32)	
SCLC, no. of patients (%)	6,748 (10.14)	337 (13.72)	
Life expectancy, mean (SE) years	1.43 (0.02)	1.44 (0.10)	0.906
Lifetime healthcare expenditures per case,			
NHI-reimbursed medical costs, mean (SE) $	12,597 (703)	15,454 (1,520)	0.105
Out-of-pocket medical costs, mean (SE) $	--	4,797 (550)	
NSCLC, no. of patients (%)	59,787 (89.86)	2,119 (86.28)	
Life expectancy, mean (SE) years	3.05 (0.01)	3.19 (0.05)	0.011
Lifetime healthcare expenditures per case,			
NHI-reimbursed medical costs, mean (SE) $	18,660 (894)	21,965 (1,364)	0.049
Out-of-pocket medical costs, mean (SE) $	--	15,807 (872)	

--: not applicable; SD: standard deviation; SE: standard error; NHI: National Health Insurance; NSCLC: non-small cell lung cancer; SCLC: small cell lung cancer.

^*^Comparison using Z-statistic.

### The QALE and loss-of-QALE

**Figure 2:**
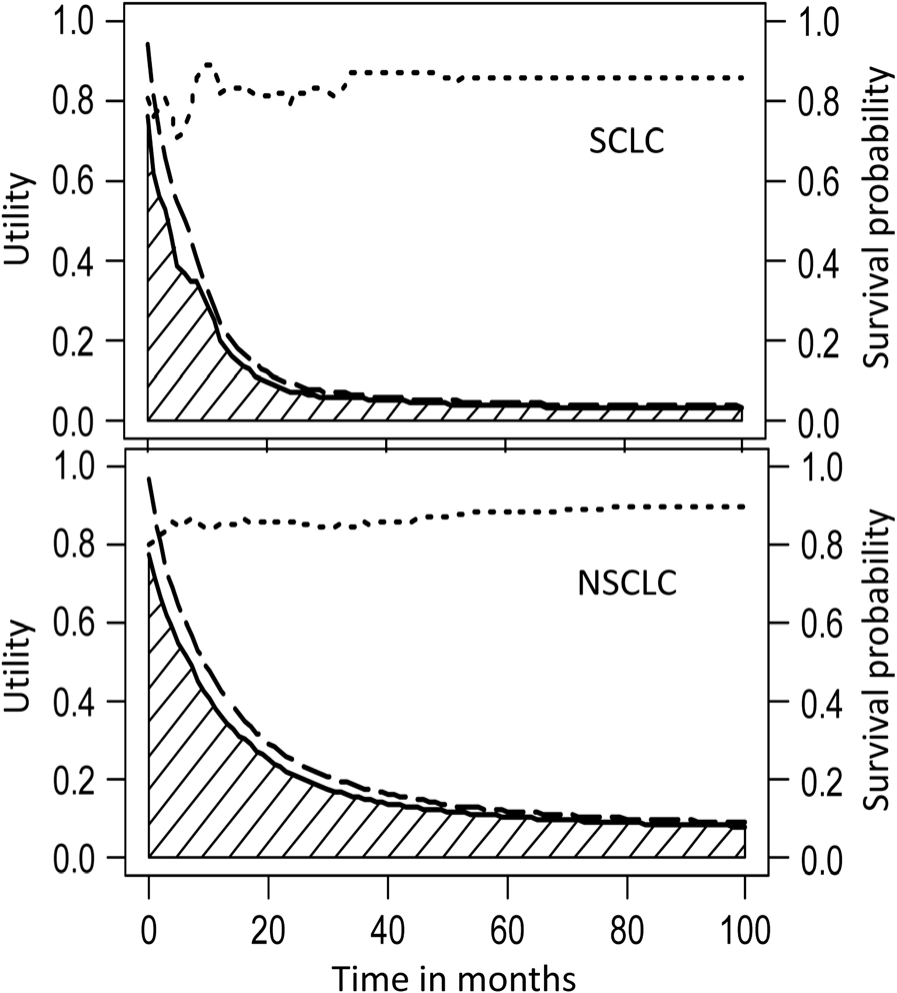
**The QALE (quality-adjusted life expectancy) of patients with small cell lung cancer (SCLC, upper panel) and non-small cell lung cancer (NSCLC, lower panel).** The survival curves (dashed lines), mean utility functions (dotted lines), and quality-adjusted survival curves (solid lines) of patients with lung cancer are depicted, and the shaded area represents the QALE.

**Figure 3:**
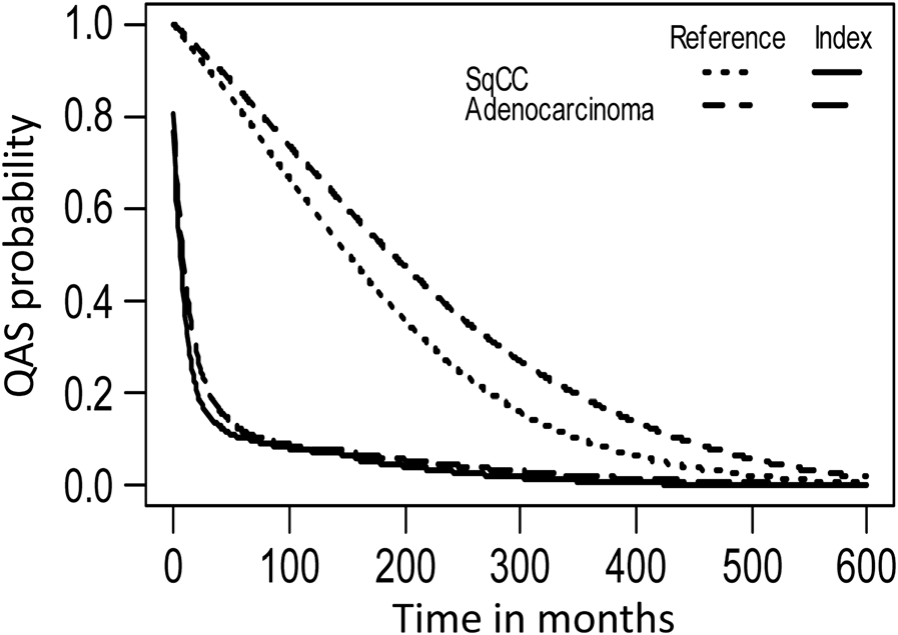
**The QAS (quality-adjusted survival) curves of patients with squamous cell carcinoma (SqCC), adenocarcinoma and the corresponding referents.** The difference between the QAS curve of patients and that of the corresponding referents was the loss-of-QALE (quality-adjusted life expectancy). SqCC patients: solid line; age- and sex-matched referents of SqCC: dotted line; adenocarcinoma patients: long-dashed line; age- and sex-matched referents of adenocarcinoma: short-dashed line.

After multiplying the survival probability with the mean QoL at each time *t* (duration-to-date), we obtained the quality-adjusted survival curve, as shown in Figure 2. The sum of the shaded area under the curve represents the QALE. Assuming that the QoL of age- and sex-matched referents was 1, the difference between the quality-adjusted survival curve of patients and that of the referents was the loss-of-QALE (Figure 3). The QALE for patients with SCLC and NSCLC was 1.21 and 2.65 QALY, respectively, while the loss-of-QALE for the two groups was 13.69 and 14.08 QALY, respectively. Compared with NSCLC patients with squamous cell carcinoma (SqCC), those with adenocarcinoma had a longer QALE (3.03 vs. 2.37 QALY, *p* < 0.001, Table 2). Moreover, we found that the loss-of-QALE of adenocarcinoma patients was significantly greater than that of SqCC patients (15.03 vs. 12.22 QALY, *p* < 0.001), probably because of the younger mean age at diagnosis (65.60 vs. 69.35 years, *p* < 0.001).

**Table 2:** The QALE (quality-adjusted life expectancy), loss-of-QALE, healthcare expenditures (in US dollars) of lung cancer patients

	**NSCLC (n = 59,787)**		
	**SqCC (n = 16,796)**	**Adenocarcinoma (n = 33,669)**	Other NSCLCs^* ^**(n = 9,322)**
Age at diagnosis, mean (SD) years	69.35 (10.63)	65.60 (12.84)	68.12 (12.11)
Sex, no. of males (%)	14,423 (85.87)	18,075 (53.68)	6,625 (71.07)
Life expectancy, mean (SE) years	2.73 (0.02)	3.46 (0.02)	1.99 (0.02)
Expected years of life lost, mean (SE) years	11.84 (0.03)	14.61 (0.03)	13.91 (0.03)
QALE, mean (SE) QALY	2.37 (0.05)	3.03 (0.03)	1.74 (0.07)
Loss-of-QALE, mean (SE) QALY			
Assumed mean utility of referents = 1	12.22 (0.05)	15.03 (0.04)	14.17 (0.08)
Assumed mean utility of referents = 0.95	11.45 (0.05)	14.16 (0.03)	13.35 (0.08)
Assumed mean utility of referents = 0.9	10.75 (0.05)	13.25 (0.04)	12.58 (0.08)
Lifetime healthcare expenditures per case,			
NHI-reimbursed medical costs, mean (SE) $	14,249 (1,435)	18,150 (1,469)	12,918 (1,156)
Out-of-pocket medical costs, mean (SE) $	6,350 (741)	18,620 (1,857)	7,439 (842)
Healthcare expenditures/life-year per case, mean (SE) $	9,281 (803)	13,636 (754)	12,546 (1,050)
Healthcare expenditures/QALY per case, mean (SE) $	10,841 (980)	15,642 (885)	14,475 (1,400)

SD: standard deviation; SE: standard error; NSCLC: non-small cell lung cancer; QALY: quality-adjusted life year; SqCC: squamous cell carcinoma.

^*^Large cell carcinoma, adenosquamous cell carcinoma, adenoid cystic carcinoma, mucoepidermoid carcinoma, carcinoid tumor and unspecified carcinoma.

### The lifetime healthcare expenditures

**Figure 4:**
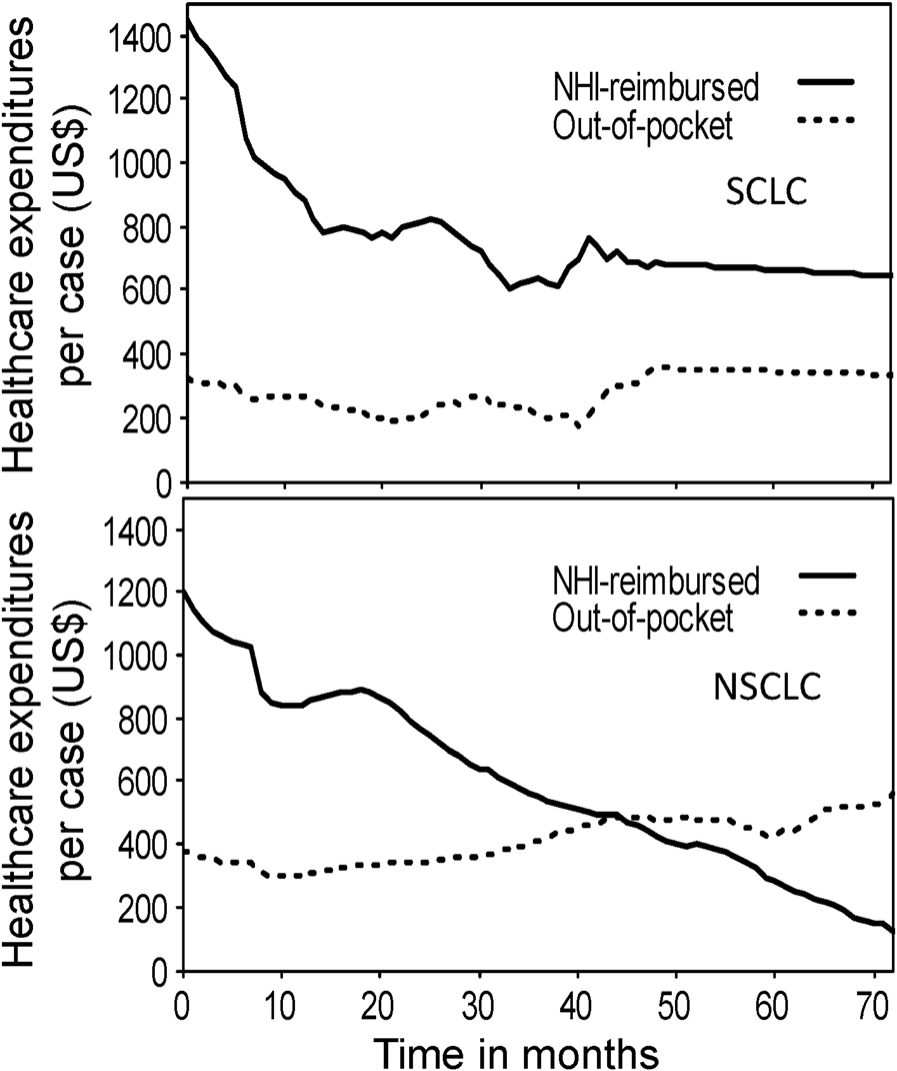
**The healthcare expenditures per case of patients with small cell lung cancer (SCLC, upper panel) and non-small cell lung cancer (NSCLC, lower panel).** The medical costs reimbursed by the National Health Insurance (NHI) are depicted in solid lines and out-of-pocket medical costs are depicted in dotted lines.

Using the kernel-smoothing method, the average healthcare expenditures per case of patients with SCLC and NSCLC are depicted in Figure 4. It shows that the medical costs reimbursed by the NHI decreased gradually after the diagnosis; however, the out-of-pocket medical costs in the terminal phase of NSCLC increased. Compared NHI-reimbursed costs of the 987 patients in our hospital with those in the national database, we found that healthcare costs of the NCKUH for SCLC and NSCLC patients accounted for 74.3% and 84.1%, respectively, of the total costs reimbursed by the NHI.

Table 2 also shows the lifetime healthcare expenditures per case for patients with different NSCLC subtypes, adjusted for medical care inflation rates up to the year 2012 and for annual discount rates in future years. Compared with SqCC patients, adenocarcinoma patients had greater lifetime healthcare expenditures per case (US$ 36,771 ± 1,998 versus US$ 20,599 ± 1,787), especially in regard to out-of-pocket medical costs (US$ 18,620 ± 1,857 versus US$ 6,350 ± 741). Moreover, we found that both the annual healthcare expenditures and cost-per-QALY attributable to adenocarcinoma were higher than those for SqCC.

### Validation of the extrapolation method

There were 4,425 patients with SCLC and 38,509 patients with NSCLC diagnosed during the first seven years, between 1998 and 2004, of which the survival curves were extrapolated to 2010 and compared with the Kaplan-Meier estimates based on the 13-year follow-up (Table 3). The relative biases of the extrapolation ranged between + 7.71% (*p* = 0.122) and + 1.29% (*p* = 0.551).

**Table 3:** Validation of the extrapolated estimates by 13 years of follow-up and the Kaplan-Meier method

	**Cohort size**	**13-year follow-up Kaplan-Meier estimate, mean (SE) months**	**Estimate using the extrapolation based on the first 7 years of follow-up, mean (SE) months**	**Relative bias,%**	***P *** value^*^
SCLC	4,425	14.75 (0.45)	15.42 (0.38)	+4.54	0.424
NSCLC	38,509	25.27 (0.24)	25.89 (0.16)	+2.45	0.165
SqCC	11,507	25.51 (0.43)	26.21 (0.31)	+2.74	0.528
Adenocarcinoma	20,926	27.05 (0.30)	27.40 (0.23)	+1.29	0.551
Other NSCLCs	6,076	18.68 (0.44)	20.12 (0.37)	+7.71	0.122

SE: standard error; NSCLC: non-small cell lung cancer; SCLC: small cell lung cancer; SqCC: squamous cell carcinoma.

^*^Comparison using Z-statistic.

## Discussion

Although the incidence of adenocarcinoma (50.60%) was about twice that of SqCC (25.24%) during the period 1998–2007, according to the data from the National Cancer Registry, the former has not attracted as much attention as the latter, which is associated with cigarette smoking [22] and has been the subject of much greater preventative efforts for the last few decades. There is a general impression that long-term survival is similarly poor with all types of NSCLC, a view that is corroborated by the results of this study, which found a life expectancy of 2.73 versus 3.46 years for patients with SqCC and adenocarcinoma, respectively (Table 2). However, after adjustment for the age at onset, the latter group suffered from 2.77 (= 14.61-11.84) additional years of life lost in comparison with the former, which was equivalent to 2.81 (= 15.03-12.22) QALY more loss-of-QALE (Table 2). Moreover, the average healthcare expenditures per life-year for patients with adenocarcinoma were 46.9% (= (13,636-9,281)/9,281) higher than that of SqCC patients, while the average lifetime healthcare expenditures were 78.5% (= (36,771-20,599)/20,599) higher. As this study is limited to cases with pathological evidence that were followed for 13 years, sex- and age-matched for estimating the life expectancy, and adjusted for the QoL and costs of an actual cohort, the estimation is not confounded by the factors listed above. In addition, the validation of our extrapolation method has shown that the relative biases were all less than 7.71% after 6 years of extrapolation (Table 3). We thus tentatively conclude that, in Taiwan, the lifelong health impact and financial burdens of adenocarcinoma are heavier than those of SqCC and deserve effort with regard to prevention.

Several risk factors for adenocarcinoma have been identified. In addition to cigarette smoking, indoor exposure to radon gas is perhaps one of the most important [23,24] and may be easily preventable. The risk of lung cancer among non-smoking women also appears to be associated with cooking fume exposure and lack of ventilation control [25-27]. By carefully studying and controlling these risk factors, more life-years and QALY lost, as well as healthcare expenses, might be saved.

Unlike previous studies that have used internationally-selected life tables together with expert determination of QoL values to calculate the disease burden of lung cancer with the disability-adjusted life year (DALY) unit [28,29], our study used the national life tables of Taiwan and a cross-sectional sample of patients for measurement of QoL to estimate the QALE and loss-of-QALE using QALY. While the DALY method makes international comparisons more feasible, the QALY unit calculated by our method can directly compare different prevention, diagnosis, and treatment strategies, and would probably be more feasible for national health policy decisions. However, the utility values of NSCLC patients reported by our cross-sectional sample were higher than those reported in other studies [30,31], and this may be due to several reasons. First, all patients must be healthy enough to accept our interview invitations and most (85.4%, 528 of 618 patients) of our subjects were recruited from outpatient departments. They were less likely to have any severe adverse effect and would have higher utility scores [30]. In addition, 59.1% (365 of 618 patients) of our subjects did not have any evidence of metastasis, which would make the average scores of our patients be higher than those with 63.2% of metastasis [31]. Second, because insight into the diagnosis of lung cancer was one of the inclusion criteria required by the Institutional Review Board, the utility values of our patients were usually higher [32]. Third, while extrapolating the QoL function to lifetime, it was assumed that patients remained at the same level of QoL near the end of the follow-up period. Such an assumption could result in an overestimated or higher QoL score because actual utility value usually declines with age [33]. Hence, the QALE would be overestimated while the loss-of-QALE would be underestimated.

The lifetime healthcare expenditures for adenocarcinoma, even if we look at the expenditures per life-year, were higher than those for SqCC. This can be at least partially explained by the fact that the patients with adenocarcinoma were 3.75 years younger than those with SqCC and usually received more aggressive forms of treatments, which included newly developed chemotherapeutic agents (such as premetrexed, gefitinib, erlotinib, and so on). Most costs of these treatments were out-of-pocket medical costs, which were administered in the later and/or terminal phase of the disease, as also shown on Figure 4. When estimating the lifetime healthcare expenditures, we adjusted the dollar values throughout the 2005–2012 period to that of 2012. In addition, the current estimates were adjusted for an annual discount rate of 3% in future years. Comparisons with studies from other countries [34,35] reveal that the lifetime healthcare expenditures reimbursed by the NHI in Taiwan seem relatively small. This could be explained by the different prices of medical care in different countries.

Several limitations must be acknowledged in our study. First, the QoL score of the reference population was assumed to be 1, whereas the reference population might include people with other illnesses and, as a result, the loss-of-QALE due to lung cancer might be overestimated. However, we have tested the assumption in sensitivity analysis and assessed its impact on the results (Table 2). In addition, since both reference groups for the two different subtypes of lung cancer were treated in the same way, the difference between them would not be confounded. Second, although the estimated lifetime healthcare expenditures represented not only the medical costs paid by the NHI but also the out-of-pocket money, there were still other expenses paid by the patients and/or their families, including transportation costs, payments made to caregivers, and human capital loss (i.e. the foregone earnings), which we did not take into consideration. Nevertheless, our estimations could be considered as a lower bound and may be useful for future lung cancer prevention programs planned by the related health authorities. Third, as the data abstracted from the National Cancer Registry for survival analysis did not have detailed information of tumor stages, we could not do further subgroup analysis regarding the staging issue. Because about three-fourth of NSCLC and all SCLC patients are inoperable, the magnitude of potential bias may not be too large. Lastly, the estimation of QALE would have been more accurate if we had obtained the QoL follow-up for every patient in the lung cancer cohort. Future longitudinal studies are indicated to corroborate our results based on cross-sectional samples.

## Conclusions

In conclusion, this study successfully estimated the QALE, loss-of-QALE, and the lifetime healthcare expenditures of patients with different lung cancer subtypes. We found that adenocarcinoma produces higher loss-of-QALE and lifetime healthcare expenditures than is seen with SqCC. Future research could focus on the cost-effectiveness of different prevention programs targeted at different types of lung cancer to obtain the cost-per-life year and/or cost-per-QALY, and provide policy recommendations. In addition, the methodology could be applied to other cancers or chronic diseases for resources planning of healthcare services.

## Abbreviations

EQ-5D: EuroQol 5-dimension questionnaire; NCKUH: National Cheng Kung University Hospital; NHI: National Health Insurance; NSCLC: Non-small cell lung cancer; QALE: Quality-adjusted life expectancy; QALY: Quality-adjusted life year; QoL: Quality of life; SCLC: Small cell lung cancer; SqCC: Squamous cell carcinoma.

## Competing interests

The authors declare that they have no competing interests.

## Authors’ contributions

SCY, WWL, JDW conceived and designed the experiments. SCY, WWL, WCS, SYW, HHWC contributed materials. SCY, WWL, YLW performed the experiments. SCY WWL MCH analyzed the data. SZY, WWL, JDW wrote the paper. All authors read and approved the final manuscript.

## Pre-publication history

The pre-publication history for this paper can be accessed here:

http://www.biomedcentral.com/1471-2407/13/579/prepub

## References

[B1] Guo P Huang ZL Yu P Li K Trends in cancer mortality in China: an update Ann Oncol 2012 23 2755 2762 10.1093/annonc/mds069 22492700

[B2] Yabroff KR Bradley CJ Mariotto AB Brown ML Feuer EJ Estimates and projections of value of life lost from cancer deaths in the United States J Natl Cancer Inst 2008 100 1755 1762 10.1093/jnci/djn383 19066267 PMC2720776

[B3] Cipriano LE Romanus D Earle CC Neville BA Halpern EF Gazelle GS McMahon PM Lung cancer treatment costs, including patient responsibility, by disease stage and treatment modality, 1992–2003 Value Health 2011 14 41 52 10.1016/j.jval.2010.10.006 21211485 PMC3150743

[B4] Drummond MF Sculpher MJ Torrance GW O’Brien BJ Stoddart GL Methods for the Economic Evaluation of Health Care Programmes 2005 3 New York: Oxford University Press

[B5] Bravo Vergel Y Sculpher M Quality-adjusted life years Pract Neurol 2008 8 175 182 10.1136/pn.2007.140186 18502950

[B6] Institute of Medicine For the Public’s Health: The Role of Measurement in Action and Accountability 2011 Washington, D.C.: The National Academies Press 24983050

[B7] Kutikova L Bowman L Chang S Long SR Obasaju C Crown WH The economic burden of lung cancer and the associated costs of treatment failure in the United States Lung Cancer 2005 50 143 154 10.1016/j.lungcan.2005.06.005 16112249

[B8] Chouaid C Atsou K Hejblum G Vergnenegre A Economics of treatments for non-small cell lung cancer Pharmacoeconomics 2009 27 113 125 10.2165/00019053-200927020-00003 19254045

[B9] Glasziou PP Cole BF Gelber RD Hilden J Simes RJ Quality-adjusted survival analysis with repeated quality of life measures Stat Med 1998 17 1215 1229 10.1002/(SICI)1097-0258(19980615)17:11<1215::AID-SIM844>3.0.CO;2-Y 9670411

[B10] Hwang JS Tsauo JY Wang JD Estimation of expected quality-adjusted survival by cross-sectional survey Stat Med 1996 15 93 102 10.1002/(SICI)1097-0258(19960115)15:1<93::AID-SIM155>3.0.CO;2-2 8614748

[B11] Hwang JS Wang JD Integrating health profile with survival for quality of life assessment Qual Life Res 2004 13 1 10 15058782 10.1023/B:QURE.0000015299.45623.38

[B12] ICD-10 Version: 2010 http://apps.who.int/classifications/icd10/browse/2010/en

[B13] Hwang JS Wang JD Monte Carlo estimation of extrapolation of quality-adjusted survival for follow-up studies Stat Med 1999 18 1627 1640 10.1002/(SICI)1097-0258(19990715)18:13<1627::AID-SIM159>3.0.CO;2-D 10407234

[B14] Fang CT Chang YY Hsu HM Twu SJ Chen KT Lin CC Huang LY Chen MY Huang JS Wang JD Chuang CY Life expectancy of patients with newly-diagnosed HIV infection in the era of highly active antiretroviral therapy Q J M 2007 100 97 105 10.1093/qjmed/hcl141 17277317

[B15] Chu PC Wang JD Hwang JS Chang YY Estimation of life expectancy and the expected years of life lost in patients with major cancers: extrapolation of survival curves under high-censored rates Value Health 2008 11 1102 1109 10.1111/j.1524-4733.2008.00350.x 18489497

[B16] Liu PH Wang JD Keating NL Expected years of life lost for six potentially preventable cancers in the United States Prev Med 2013 56 309 313 10.1016/j.ypmed.2013.02.003 23428566

[B17] Hwang JS iSQoL: integration of Survival with Quality of Life http://www.stat.sinica.edu.tw/isqol/

[B18] The EuroQol Group EuroQol - a new facility for the measurement of health-related quality of life Health Policy 1990 16 199 208 10109801 10.1016/0168-8510(90)90421-9

[B19] Szende A Oppe M Devlin N EQ-5D Value Sets: Inventory, Comparative Review and User Guide 2007 Dodreht, the Netherlands: Springer

[B20] Dolan P Modeling valuations for EuroQol health states Med Care 1997 35 1095 1108 10.1097/00005650-199711000-00002 9366889

[B21] Directorate-General of Budget, Accounting and Statistics, Executive Yuan, R.O.C. (Taiwan) http://eng.dgbas.gov.tw/mp.asp?mp=2

[B22] Boyle P Levin B World Cancer Report 2008 2008 Geneva: WHO Press

[B23] Krewski D Lubin JH Zielinski JM Alavanja M Catalan VS Field RW Klotz JB Letourneau EG Lynch CF Lyon JL Sandler DP Schoenberg JB Steck DJ Stolwijk JA Weinberg C Wilcox HB A combined analysis of North American case–control studies of residential radon and lung cancer J Toxicol Environ Health A 2006 69 533 597 10.1080/15287390500260945 16608828

[B24] Darby S Hill D Deo H Auvinen A Barros-Dios JM Baysson H Bochicchio F Falk R Farchi S Figueiras A Hakama M Heid I Hunter N Kreienbrock L Kreuzer M Lagarde F Mäkeläinen I Muirhead C Oberaigner W Pershagen G Ruosteenoja E Rosario AS Tirmarche M Tomásek L Whitley E Wichmann HE Doll R Residential radon and lung cancer - detailed results of a collaborative analysis of individual data on 7148 persons with lung cancer and 14,208 persons without lung cancer from 13 epidemiologic studies in Europe Scand J Work Environ Health 2006 32 Suppl 1 1 83 16538937

[B25] Ko YC Lee CH Chen MJ Huang CC Chang WY Lin HJ Wang HZ Chang PY Risk factors for primary lung cancer among non-smoking women in Taiwan Int J Epidemiol 1997 26 24 31 10.1093/ije/26.1.24 9126500

[B26] Ko YC Cheng LS Lee CH Huang JJ Huang MS Kao EL Wang HZ Lin HJ Chinese food cooking and lung cancer in women nonsmokers Am J Epidemiol 2000 151 140 147 10.1093/oxfordjournals.aje.a010181 10645816

[B27] Metayer C Wang Z Kleinerman RA Wang L Brenner AV Cui H Cao J Lubin JH Cooking oil fumes and risk of lung cancer in women in rural Gansu, China Lung Cancer 2002 35 111 117 10.1016/S0169-5002(01)00412-3 11804682

[B28] Murthy NS Nandakumar BS Pruthvish S George PS Mathew A Disability-adjusted life years for cancer patients in India Asian Pac J Cancer Prev 2010 11 633 640 21039029

[B29] Pham TM Kubo T Fujino Y Ozasa K Matsuda S Yoshimura T Disability-adjusted life years (DALY) for cancer in Japan in 2000 J Epidemiol 2011 21 309 312 10.2188/jea.JE20110017 21628841 PMC3899425

[B30] Grutters JPC Joore MA Wiegman EM Langendijk JA de Ruysscher D Hochstenbag M Botterweck A Lambin P Pijls-Johannesma M Health-related quality of life in patients surviving non-small cell lung cancer Thorax 2010 65 903 907 10.1136/thx.2010.136390 20861294

[B31] Trippoli S Vaiani M Lucioni C Messori A Quality of life and utility in patients with non-small cell lung cancer Pharmacoeconomics 2001 19 855 863 10.2165/00019053-200119080-00007 11596837

[B32] Lee LJ Chung CW Chang YY Lee YC Yang CH Liou SH Liu PH Wang JD Comparison of the quality of life between patients with non-small-cell lung cancer and healthy controls Qual Life Res 2011 20 415 423 10.1007/s11136-010-9761-y 20953907

[B33] Luo N Johnson JA Shaw JW Feeny D Coons SJ Self-reported health status of the general adult U.S. population as assessed by the EQ-5D and Health Utilities Index Med Care 2005 43 1078 1086 10.1097/01.mlr.0000182493.57090.c1 16224300

[B34] Kahende JW Woollery TA Lee CW Assessing medical expenditures on 4 smoking-related diseases, 1996–2001 Am J Health Behav 2007 31 602 611 10.5993/AJHB.31.6.5 17691873

[B35] Mariotto AB Yabroff KR Shao Y Feuer EJ Brown ML Projections of the cost of cancer care in the United States: 2010–2020 J Natl Cancer Inst 2011 103 117 128 10.1093/jnci/djq495 21228314 PMC3107566

